# Understanding nurses’ justification of restraint in a neurosurgical setting: A qualitative interview study

**DOI:** 10.1177/09697330221111447

**Published:** 2022-10-20

**Authors:** Amina Guenna Holmgren, Ann-Christin von Vogelsang, Anna Lindblad, Niklas Juth

**Affiliations:** 27106 Karolinska Institutet, Stockholm, Sweden; 59562 Karolinska University Hospital, Stockholm, Sweden; 59562 Karolinska University Hospital, Stockholm, Sweden; 97092 Karolinska Institutet, Stockholm, Sweden; 27106 Karolinska Institutet, Stockholm, Sweden; 8097 Uppsala University, Uppsala, Sweden

**Keywords:** Ethics, justification, neurosurgery, restraint, autonomy, nursing

## Abstract

**Background:**

Despite its negative impact on patients and nurses, the use of restraint in somatic health care continues in many settings. Understanding the reasons and justifications for the use of restraint among nurses is crucial in order to manage this challenge.

**Aim:**

To understand nurses’ justifications for restraint use in neurosurgical care.

**Research design:**

A qualitative, descriptive design was used. Data were analysed with inductive qualitative content analysis.

**Participants and research context:**

Semi-structured interviews with 15 nurses working in three neurosurgical departments in Sweden.

**Ethical considerations:**

Approved by The Regional Ethics Committee, Stockholm, Sweden.

**Findings:**

The analysis resulted in three categories. The category *Patient factors influencing restraint use* describes patient factors that trigger restraint, such as a diminished decision-making competence, restlessness, and need for invasive devices. The category *Specific reasons for justifying restraint* describes reasons for restraining patients, such as restraint being used for the sake of the patient or for the sake of others. The category *General reasoning in justifying restraint* describes how nurses reason when using restraint, and the decision to use restraint was often based on a consequentialist approach where the nurses’ weighed the pros and cons of different alternatives.

**Discussion:**

Nurses with experience of restraint use were engaged in a constant process of justifying and balancing different options and actions. Restraint was considered legitimate if the benefit exceeded the suffering, but decisions on which restraint measures to use and when to use them depended on the values of the individual nurse.

**Conclusion:**

How nurses reason when justifying restraint, why they use restraint, and who they use restraint on must be considered when creating programs and guidelines to reduce the use of restraint and to ensure that when it is used it is used carefully, appropriately, and with respect.

## Introduction

Respect for patients’ autonomy and giving care based on patients’ needs and wishes is a fundamental part of nursing. Another important principle is to provide safe care with the aim of doing good.^1^ However, it is not always easy to obtain both, and in certain situations the principles may even be in conflict. An example of this is when patients refuse the care required to maintain or restore health. In such situations, healthcare professionals may choose to force the patient to comply with treatment by different sorts of restraint.

Physical restraint can be defined as any procedure or action that prevents free body movement to a position of choice and/or normal access to his or her body with the use of a method that he or she cannot easily remove.^2^ Examples of physical restraint include, but are not limited to, hand mittens, sheet ties, wrist and ankle ties, belts, and restrictive side rails.^3^ Another form of restraint is chemical restraint. Chemical restraint is the use of psychotropic medication to control the patient.^3^ In this paper, the term restraint will be used for both physical and chemical restraint unless otherwise explicitly stated.

Restraint and its implications for health care have been studied and debated for decades; nevertheless, there are areas within somatic care which have been overlooked. For instance, patients in neurosurgical care are at a high risk of being subjected to restraint.^4,5^ It has been reported that 6–16% of the patients at a neurosurgical department were subjected to restraint daily, but studies concerning reasoning about restraint for this group of patients are lacking.^6^ It is therefore not known whether there are any differences between patients cared for at neurosurgical departments compared to other healthcare settings such as intensive care and geriatric care. Patients with injuries or diseases of the central nervous system may be cognitively affected and their decision-making capacity impaired. Communicating medical information and consent to treatment may therefore be a challenging task for healthcare professionals.^7^

Nurses are often the primary decision-makers when restraint is used in somatic care, and this has been described as an unwanted responsibility.^8,9^ An interplay of different contextual factors has been described to affect the decision-making process, for instance, the legal context, the working environment, and nurses’ relationships with colleagues, patients, and patients’ families.^10^

In previous research, nurses have reported decisions on restraint to be difficult, both from a practical and an emotional perspective.^8,11^ The practical problems include uncertainty regarding how and when to make the decision and how to execute the restraint.

Restraint in intensive care and geriatric care is often justified by the argument that the patient must be protected from him/herself.^12,13^ However, restraint aimed at protecting patients from harm may be ineffective or even counterproductive because it can result in a range of serious negative consequences associated with both physical harm (such as pressure ulcers, falls, and even death) and psychological harm.^14,15^ It has also been reported that patients previously subjected to restraint are more reluctant to seek help at emergency departments for new health problems.^16^

Despite its negative impact on patients and nurses, the use of restraint continues. Increased knowledge on nurses’ justification for using restraint in specific contexts is vital in order to gain a deeper understanding of the phenomenon and to be able to evaluate the possibilities to reduce the use of restraint. Considering the lack of studies concerning nurses’ justification for restraint in neurosurgical settings, the aim of this study was to understand how nurses justify the use of restraint in neurosurgical care.

## Methods

### Research design

A qualitative, descriptive design guided by a naturalistic inquiry (i.e., to inductively study a phenomenon in its natural state with no a priori commitment to any theoretical view) was used to examine nurses’ justification for restraint. The naturalistic inquiry approach can be viewed as a commitment to describe experiences from a context where the phenomenon naturally occurs, in contrast to for instance grounded theory or ethnographic studies, which are based on more strict methodological frameworks.^17^ The qualitative descriptive design is well suited to study individuals’ experiences when aiming to give descriptions of a phenomenon.^17^ The Consolidating Criteria for Reporting Qualitative Research checklist was used to ensure the quality reporting of this study.^18^

### Participants and research context

The study was conducted at three of Sweden’s six neurosurgical departments. All three departments care for patients with similar diagnosis and are similarly organised (a neurointensive care unit, an intermediate care unit, and inpatient wards). All in all, they serve half of Sweden’s population with specialised neurosurgical care. A typical case sample was used. The purpose of a typical case sample is to highlight or illustrate what is typical or average, not to make generalised statements about the experiences of all respondents.^19^ The main criteria for inclusion in the study were that the respondents had experience of using or deciding on restraint at a neurosurgical department. Therefore, all nurses, working at the three departments and from all units were eligible for inclusion, approximately 220 nurses. Information about the study was sent by email. The email addresses were obtained from an administrator at each department. In the email it was specified that only nurses with experience of using and/or deciding on restraint could participate in the study. Nurses with this experience and who were interested in partaking were asked to contact the administrator, who then set up the interviews. All nurses who contacted the administrator agreed to take part in the study and went through with the interview. A total of 15 interviews were conducted, five at each department.

### Data collection

Semi-structured individual interviews guided by an interview guide were performed by the first author between November 2017 and May 2018. The respondents received study-specific information before the interviews and were informed that they would be asked questions about situations where they had used restraint. During the interviews, the respondents were asked to describe specific situations where they had used or taken part in the decision to use restraint. Probing questions about the decision-making process and reasons for restraining the patient were also asked (Appendix 1).

The interview guide was developed and evaluated by the researchers: two nurses with experience of neurosurgical care and restraint use, a psychiatrist with experience of restraint and compulsory care in psychiatry, and an ethicist with theoretical knowledge regarding compulsory care. The interview guide was piloted at one of the departments before the study but did not result in any changes. The interviews were performed at the respondents’ workplace in a secluded room. All interviews were audio recorded and transcribed verbatim.

### Data analysis

The interview material was analysed with inductive conventional content analysis according to Hsieh and Shannon.^20^ This type of analysis is suitable when the aim of a study is to elucidate participant experience rather than to investigate any deeper meaning of the phenomenon in question. The transcribed interviews were read repeatedly by at least two of the authors to obtain a sense of the whole. Words and sentences that appeared to capture key statements or concepts related to the aim of the study were then highlighted and coded.

Codes sharing similar features were clustered, followed by a preliminary interpretation of the codes, and sorting into subcategories. The subcategories were reassessed by rereading the interviews and comparing them to the codes and subcategories. This was done to ensure that the meaning of the interview text corresponded with the codes and subcategories. After this step, the subcategories were organised into more general categories. Lastly, definitions for each subcategory and category were developed, in order to ensure that the categories were externally heterogeneous and internally homogeneous.^21^ The authors assessed that data saturation was reached, and no additional aspects of the phenomenon emerged when analysing the last interviews.^22^

### Ethical considerations

A regional ethics committee in Sweden approved the study (2017/1215/-31/5). The respondents received written and oral information about the study and only participated after signing written consent. They were informed about their right to withdraw from the study at any time without explanation. Furthermore, they were informed that the information shared would not be forwarded to their employers and that the information would be handled confidentially. The transcribed interviews were pseudonymised with interview-specific code numbers to ensure that identification of interviewees was not possible. If staff members were mentioned during the interviews, names were omitted. Code lists were locked up separated from the collected data. All data collection was carried out without dependencies in the relationships between the researcher and the participants. All data were handled with respect for confidentiality, and the interview material was stored in a secure place. Only researchers in the project had access to uncoded material.

### Rigour

Several strategies were used to ensure trustworthiness. Credibility and confirmability were enhanced by using investigation triangulation.^23^ The first author read and analysed all 15 interviews, whereas the other authors read and analysed five interviews each. All authors read and analysed the material separately and then met at several occasions to discuss and compare their analysis, and discussion continued until consensus was reached and all authors agreed on the analysis. This was done to prevent researcher bias and to ensure that subcategories and categories were as externally heterogeneous and internally homogenous as possible.^21^ Dependability was strengthened by using an interview guide ensuring a consistent data collection. One author (the first author) conducted all interviews during a limited period. The analysis processes followed the chosen design. To increase the results’ transferability, three out of Sweden’s six neurosurgical departments were included in the study, thus ensuring that the results did not reflect any specific department’s culture.

## Results

The interviews lasted 20–50 min, with an average of 30 min. Characteristics of the respondents are presented on the group level in order not to reveal any identities; see Table 1.

**Table 1.** Characteristics of respondents (*n* = 15).

Characteristics	Numbers
Age (years)	
20–29	0
30–39	6
40–49	1
50–59	6
60–69	2
Sex	
Male	3
Female	12
Experience as a nurse (years)	
0–4	1
5–9	3
10–14	2
15–19	1
20–24	1
25–29	3
30–35	4
Respondents’ workplaces^a^	
Neurointensive care unit	6
Intermediate care unit	12
Neurosurgical ward	7

^a^Some nurses worked at more than one unit; the sum of the respondents’ workplaces therefore exceeds the total number of respondents.

**Figure 1.**
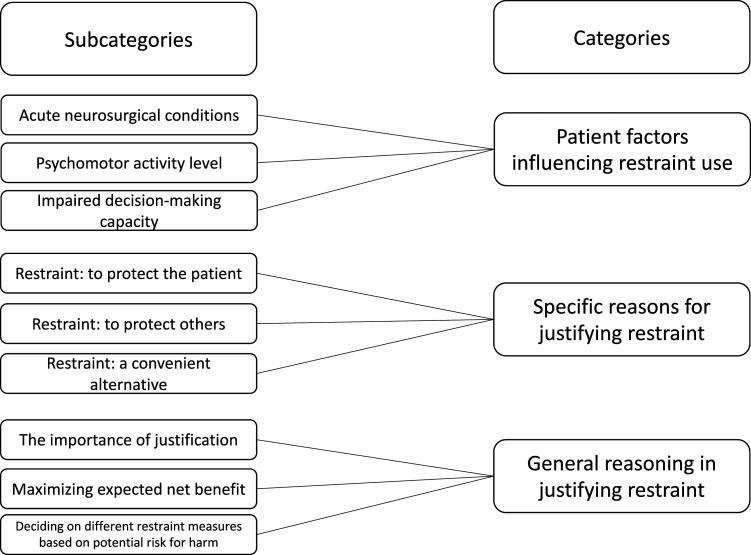
Subcategories and categories.

The analysis of the textual data resulted in three categories and nine subcategories as presented in Figure 1. Examples of quotes are given in Table 2.

**Table 2.** Examples quotes, codes, subcategories, and categories (quotes presented with respondents’ code numbers).

Quote	Code	Subcategory	Category
Most often patients with Subarachnoid haemorrhages […] It is mostly that group of patients, that I can think of right now. (Respondent 8)	Subarachnoid haemorrhage	Acute neurosurgical conditions	Patient factors influencing restraint use
He was completely disoriented when he woke up, but he was very strong, started acting out, and started kicking. […] he was both verbally and physically threatening (Respondent 7)	Disoriented and acting out	Psychomotor activity level	
They do not understand, have a lacking insight, they can count, they can write, they can read, but they do not understand what they write or read. Their sense of logic is gone, they remember things from the past, many wants to go to work or do something, and they do not understand that they cannot [do]. (Respondent 1)	Impaired memory and insight	Impaired decision capacity	
It often feels like one do it to protect them, not to harm them (Respondent 13)	Restraint to protect and not to harm	Restraint: to protect the patient	Specific reasons for justifying restraint
It was a lot of work, and I had other patients to take care of. Felt that if she disappears, I do not know what happens, felt that I almost had to [restrain]. (Respondent 3)	Had other patients to care for	Restraint: to protect others	
There are probably some [colleagues] who have the same feelings as me, I think. While others just like ‘Ah, put on this hand mitten so we get some peace and quiet here’ (Respondent 17)	Hand mittens for the sake of the healthcare staff	Restraint: a convenient alternative	
[…] justify your actions, so that if the patient can actually remember what happened and reports it afterwards and it is important that you can defend yourself. (Respondent 5)	Defend your actions	The importance of justification	General reasoning in justifying restraint
Of course, it is not good but at the same time, if you do not use it [restraint], the consequences can be so much bigger and worse. They may need surgery or not even survive. (Respondent 3)	Major consequences if restraint is not exercised	Maximising expected net benefit	
For me, it’s uncomfortable to grab someone with force to pull down someone’s arm or hand. It feels uncomfortable. For me, for some reason it feels easier to give medication. (Respondent 4)	Easier to give medication	Deciding on different restraint measures based on potential risk for harm	

### Patient factors influencing restraint use

The category consists of three subcategories and describes patient factors that triggered the use of restraint. These contextual factors influenced the nurses’ decision-making and were used as justifications for the use of restraint.

#### Acute neurosurgical conditions

According to the nurses, patients who were subjected to restraint were often treated for acute neurosurgical conditions the most common being subarachnoid haemorrhage and traumatic head injuries.

Interviewer: Which are the most common diagnoses that the [restrained] patients suffer from?

Respondent: Trauma and subarachnoid haemorrhage (Respondent 4)

The patients were described as neurologically affected and seriously ill. According to the nurses, the patients needed invasive devices and drainage for their survival such as central lines, arterial lines, tracheostomy tubes, feeding tubes, external ventricular drains, and surgical drains.

#### Psychomotor activity level

Patients were described as restless and pulling at their invasive devices and drainages. Agitation and anxiety often occurred while regaining consciousness and were described to fluctuate during the day. Furthermore, patients were perceived to be bothered by the invasive devices and drainages and therefore tried to pull them out.

Sometimes he was kind and sometimes he was mean. But then he became very worried and wanted to get up and away from here and took no pills and was aggressive towards everyone who came close to him. So then we used restraint. We had to hold on to each arm and each leg and give him first subcutaneous medicine and then put a peripheral venous catheter to start an infusion with drugs. (Respondent 6)

#### Impaired decision-making capacity

The restrained patients were described as confused and lacking insight, with decreased ability to understand information and to make their own informed decisions. One nurse portrayed the patients as being in twilight, a state neither awake nor unconscious.

Probably because they are in what I usually call the twilight land, that they are not fully awake even though they may seem completely awake but they are not, and they don’t have insight about their condition. They are simply so affected by their disease. (Respondent 11)

Other nurses gave similar reports: patients being confused and barely conscious, but at the same time physically active – a state where they could be dangerous to themselves. That patients did not understand their own best interests, and that it was difficult to assess how much the patients understood were common features of the respondents’ narratives.

Even patients who appeared to have the ability to make informed decisions lacked insight at times. Also, patients had difficulty understanding what they were experiencing and why they had invasive devices, no matter that healthcare staff repeatedly tried to explain this. Although patients had been informed before surgery, many woke up after surgery confused. The respondents also experienced that the patients had difficulty communicating their needs and wishes.

### Specific reasons for justifying restraint

The category describes reasons for applying and using restraint and consists of three subcategories. The justifications ranged from restraint for the patients’ well-being to restraint for the staffs’ convenience.

#### Restraint: To protect the patient

The main reason given by the nurses as justification for using restraint was to protect the patient, that is, restraint was used to prevent patients from harming themselves. For instance, situations where patients tried to pull out invasive devices were described (such as central lines, arterial lines, tracheostomy tubes, and feeding tubes) and important drainages (such as external ventricular drains and surgical drains), and nurses reported that it was crucial to stop patients from doing so in their own best interest.

It is for their sake, to save their brains and bodies that one does it. (Respondent 5)

The act of using restraint became a method for treating and caring for the patients, and the purpose of restraint was to help to promote and restore the patients’ health. The nurses argued that by preventing patients from pulling out vital equipment, which may have negative or even lethal consequences, the patients’ chances of recovery were improved.

#### Restraint: To protect others

Restraint was not only used to protect the individual patient, but also to protect others. Restless and aggressive patients were described as a hindrance to giving other patients the care they needed. Restraint was therefore seen as a method of controlling and helping healthcare professionals to care for other patients. The respondents expressed that they did not have the time to be constantly bedside, which the patients would have required if they were not restrained.

Perhaps one could be even more careful, all the time, and never ever stop looking at the patient. But it does not work, you have to chart, you have to take care of other patients and so on. (Respondent 12)

Furthermore, restraint was used a method to protect the healthcare staff when caring for aggressive patients. Patients exposed healthcare staff to both verbal and physical threats. The latter was exemplified by patients hitting and kicking members of the staff.

#### Restraint: A convenient alternative

Respondents described that restraint was sometimes used because it was considered convenient for the staff, that is, it was not applied to benefit the patient, but to facilitate the working situation. By using restraint, staff members could achieve some peace and quiet and allow nurses to perform multiple clinical tasks.

The times when the patient benefits in some way and the times when it may also be a little easier to be the staff in a room with a restless patient. (Respondent 7)

None of the nurses discussing restraint for the benefit of healthcare staff talked about themselves in that context, only of what their colleagues did.

### General reasoning in justifying restraint

The category describes nurses’ reasoning when justifying restraint and thereby weighing the moral costs of it and consists of three subcategories. The decision to use restraint was often based on a consequentialist approach where the nurses weighed consequences for the different actions against each other, i.e. the benefits were weighed against the presumed suffering.

#### The importance of justification

Respondents described that it was important to justify the restraint, not only for the patients’ sake, but also for their own sake. If restraint could be justified in an acceptable way, it felt better to carry it out, the respondents argued. They also described that being able to justify the restraint was important in case they would have to defend the decision. Furthermore, they argued that if restraint could not be justified, it should not be used.

The respondents viewed restraint as a last resort and something that was decided upon when no other alternatives remained. Several even described it as a necessary evil, voicing a desire to avoid restraint if able to find another solution.

While I somehow accepted that we have tried what we can, and that this is the best for the patient, even if it does not feel that way to me. I know that there is not much else that we can do. (Respondent 7)

#### Maximising expected net benefit

The nurses were engaged in a constant process of balancing and assessing risks and presumed consequences of various actions when deciding on restraint use, and the benefits of using restraint were weighed against the suffering it may cause in each individual situation. In their descriptions of situations where restraint had been used, patients trying to remove invasive devices were often mentioned. The nurses voiced concerns about the consequences for pulling out invasive devices, especially the risk of patients needing surgery. Surgery was considered a major intervention and a greater danger than, for instance, having the patients’ hands wrapped or administering sedatives. The nurses argued that the far-reaching consequences of patients pulling out invasive devices outweighed the short-term suffering of restraint. Medical safety was thus given a higher priority than the patients’ integrity. For the restraint to be ethically justified, the nurses argued that the benefit of a measure needed to exceed the suffering that it may cause.

If the benefit exceeds the suffering for the patient, it is an action we take, because we think this is for the benefit of the patient. (Respondent 12)

#### Deciding on different restraint measures based on potential risk for harm

The process of deciding on which restraining method to use depended on the values and preferences of each individual nurse, but also of the clinical situation. In the interviews, it became clear that the respondents had different preferences concerning restraint measures. However, if the preferred measure did not have the desired effect, other measures were used. Respondents described choosing the measure least harmful for the patient; however, there was no consensus on which type of method would be less harmful. During analysis, a division between physical restraint and chemical restraint emerged. Some of the nurses argued that physical measures, such as hand mittens, were more restrictive than giving sedatives, whereas others argued the opposite.

Not morally right to use hand mittens, it's too much and too rough. Like a form of abuse in a way. (Respondent 3)

The respondents also mentioned physical restraint measures which they considered more serious and wrong than others. Tying a patient down was described by several nurses as indefensible and wrong, whereas hand mittens were considered more acceptable.

Nurses preferring physical restraint to chemical restraint justified their position by arguing that sedation was more dangerous than holding a patient. They described risks associated with sedatives. Respondents who preferred chemical restraint, on the other hand, argued that holding down a patient or using hand mittens was a greater abuse than giving sedatives because the patient would be aware of what was happening. The respondents stated that physical measures were more apparent and visible both for themselves but mainly for the patients. They also described that some of the restrained patients tried to remove the hand mittens or break free when held down by staff members. Particularly aggressive patients were described as becoming more aggressive when physically restrained.

He became even wilder, even more verbal and he even tried to spit on us. (Respondent 5)

One nurse believed sedation to be easier since it would prevent staff from getting into a hand-to-hand fight with the patient, and another nurse argued that sedatives made the patient calmer and therefore more inclined to accept care. Physical measures such as hand mittens were also perceived as more restrictive since they prevented free movement. On the other hand, nurses who preferred physical restraint, believed chemical measures to be more restricting while affecting the patients’ level of consciousness.

I think it's more disturbing when you have to sedate them for it to work, because then they are not there. The hand mittens feel a bit… they can often accept that, because it's just a mitten. It does not become as conspicuous as sedation, and they can still participate […] But they still have some function with the hands with the mittens. They are not completely locked in. Sedating them is worse, if you do not find something that only calms them down a little. (Respondent 13)

## Discussion

In this study, nurses’ justification of restraint in neurosurgical care appears to be in line with research from other healthcare settings where patients with cognitive deficiency or reduced decision-making capacity are cared for. When using restraint in neurosurgical care, nurses are engaged in a constant process of justifying and balancing different options and actions. The main reason for using restraint was a desire to protect the patient from harm. Nurses’ dominant focus on safety and controlling negative behaviour has been noted before.^24^ However, the type of safety issues seems to differ between care contexts; for example, in geriatric care restraint is mostly used to stop patients from falling, whereas in intensive care, it is used to prevent treatment interference such as extubating themselves.^9,25–27^ One coping strategy in handling this is to use restraint.^28^ The latter is in accordance with the results of the present study in neurosurgical care, where restraint was reported to be used to safeguard therapeutic treatments, for instance, by stopping patients from pulling out invasive devices.

It was also reported that restraint was used to protect healthcare staff and other patients. Here, the reasons ranged from protecting staff from aggressive and violent patients to using it because they lacked staff resources. Caring for confused and sometimes aggressive patients was described to be time-consuming, which forced the nurses to prioritise between patients. This is in line with previous results, where organisational factors such as lack of time and resources have been reported to affect nurses’ use of restraint in intensive and geriatric care.^10,12,24^ Furthermore, a difficult working environment is a hindering factor for nurses in ethical decision-making and ethical action.^29^

Safety of the patient and/or safety of others was often referred to when justifying restraint, but nurses also highlighted that restraint may be used for the convenience of the staff. Interestingly, when the respondents talked about themselves, a wish to protect the patient was stated, but when they described reasons why colleagues used restraint, convenience for the healthcare staff was also mentioned. One reason for this could be a widespread view that the moral right reason for using restraint in healthcare is for the patient’s safety, and not for the convenience of healthcare staff. A systematic review on nurses’ attitudes to restraint found that nurses used restraint regardless of the moral conflict and used different coping strategies to handle their inner conflicts.^30^ Furthermore, the difference between how nurses talked about themselves, compared to others may be interpreted as an example of cognitive dissonance, since it simply cannot be the case that such justifications are only witnessed in others and never in oneself. When inconsistencies between inner values and actual behaviours arise, we may respond with feelings of discomfort, and conjuring up acceptable justifications for the behaviour may be a way to handle this discomfort.^31^ It is known that caring for patients who are in a state of confusion can be difficult and draining, and nurses have reported that it is frustrating when patients do not remember what they are told or do not follow instructions.^8,32^ Restraint is one way nurses deal with this challenge in order to establish a functioning working environment for the nursing staff.^12^ Therefore, when creating guidelines to prevent restraint, improvements of the working environment should be taken into account. Also, giving nurses tools to care for confused patients may help them avoid resorting to using restraint when they find their working environment poor.

The extent to which nurses use restraint is influenced by different factors, such as education, legislation, and guidelines.^10^ Regulation concerning restraint in somatic health care differs around the globe, ranging from forbidden to free to use. However, even in countries like Sweden, where restraint in somatic care is largely prohibited, it is still used, and the results from the present study are in line with results from studies in countries with more permissive legislation.^24,32^ This indicates that the decision-making process regarding restraint is governed by more than just legislation. Nurses rely on medical decisions, personal values, and the consequences of their actions when justifying their decisions in health care.^29^ In the present study, the justification of restraint was based on a consequentialist approach where the alternative with the least negative consequences was chosen. This is in line with previous research, where nurses weighed different options and actions against each other when deciding on restraint.^24^ Furthermore, we found that nurses prioritised certain values, where reducing or avoiding suffering were the most important in the decision-making process. The nurses were aware that restraint can create suffering, but nevertheless argued that refraining from it could create even greater suffering. Alleviating suffering is a core task for nurses and forms the very foundation of caring.^1,33^ When nursing care is considered essential for the patients well-being it is given no matter if the patient consents to it or not.^34^ Restraint has even been described as an ordinary nursing intervention and an integral part of nursing.^30^ In addition to reducing suffering, the respondents also described that the goal and purpose of using restraint was to help patients restore health. To avoid patient harm and promote patient health are uncontroversial norms in care ethics.^1,35^ Moreover, restraint was considered a means to achieve restored health and – indirectly – stronger autonomy. Side-stepping the patients’ decision in particular instances to promote the patients’ long-term autonomy has been ethically debated.^12,36^ Although this argument for paternalism was used by the respondents, autonomy was also considered important. For instance, many respondents expressed that restraint was primarily used for patients with diminished decision-making capacity, making the restraint measures into soft rather than hard paternalism. However, respondents also voiced concerns about assessing how much the patients actually understood. Decision-making competence is difficult to assess, especially since it has been described not to be a question of dichotomy but rather a question of degree. Patients may be decision-making competent in some aspects of life, but not in others.^37^

How restraint is executed depends on individual nurses’ assessments in particular situations rather than on a well-founded and common understanding on how to use restraint. This in turn results in a lack of consistency.^8,9,12^ Attitudes toward restraint use, as well as what type of restraint measure to be preferred, differ between nurses.^12^ In our study nurses’ views on different types of restraint determined which measures they primarily choose. The respondents made a distinction between physical and chemical restraint, where the latter would lower the level of consciousness by means of medication. During analysis of descriptions and arguments a difference emerged regarding which biomedical ethical principle(s) the nurses indirectly gave the highest priority. Some nurses prioritised the principle of do no harm, and others the principle of autonomy. Among the latter, physical restraint was regarded as a better alternative, since it would not alter the patient’s level of consciousness and would be more visible to the patient. The nurses prioritising the principle of do no harm, on the other hand, preferred chemical restraint by arguing that physical restraint would cause greater suffering and be more harmful since the restraint measures would became more visible to the patients. However, one may argue that hidden restraint is a risk on its own, since it excludes any insight from either patient, relatives or other healthcare professionals, thus putting patient safety at risk. The process of carrying out open restraint measures typically leaves room for patients to express their views, to invoke applicable rights, and potentially also to appeal the measure.

Identifying factors triggering restraint can be an important tool for preventing future events. In the present study, nurses’ justifications entailed statements and descriptions of the exposed patients, which add further clues regarding such factors in neurosurgical care. For instance, patients subjected to restraint were described as seriously ill, with a low level of decision-making capacity and with a need for invasive devices. It is known that patients in neurosurgical intensive care are at high risk of being exposed to restraint.^38^ More specifically, patients with traumatic brain injury and subarachnoid haemorrhage have been reported to be exposed to restraint to a large extent.^38^ According to the respondents in the present study, waking up from sedation was a phase where restraint was frequently used, which calls for guidelines and clinical training to concentrate on this time period.

Restraint use in health care is multidimensional and should be addressed in more than one way. Studies have shown that multicomponent interventions change human behaviour better than single component interventions do.^39^ Our study points out several aspects that could be included in restraint reduction programs, such as understanding contextual patient factors to prevent and treat confusion and disturbing behaviour, improving work environment, and focusing on nurses' values and attitudes concerning restraint use. Furthermore, multicomponent interventions that aim to change components at organisation’s level and address local structures at the wards have proven to be successful to reduce restraint.^40^

### Strengths and limitations

The chosen method proved to be fruitful in achieving the aim of the study. To increase the transferability interviews were conducted at three of Sweden’s six neurosurgical departments. Similar codes were found at the three departments indicating that the results did not reflect only one department’s culture but were describing nurses’ general justification of restraint in neurosurgical care. Also, the overall results were in accordance with other studies from other care contexts implying that the results can be applied to other healthcare settings.

A limitation of the study is the sensitive subject covered. Getting people to talk about things that they themselves find difficult can be challenging and respondents may refrain from describing what is considered wrong. The researchers were aware of this difficulty and tried to address it by asking questions about respondents own use of restraint as well as other nurses’ did. Participants were also carefully informed that information they provided could not be traced back to them.

## Conclusion

Nurses using restraint report being engaged in a constant process of justifying and balancing different options and actions. Foremost, restraint was considered legitimate if the expected benefit exceeded the potential suffering. However, the decision on when and what type of restraint measure to use depended on the values of the individual nurse. Also, restraint performed by others was sometimes described as a convenient alternative for healthcare professionals. Knowledge of why nurses use restraint, how they reason about it and who they use it on can support its prevention and reduction, but also ensure that when carried out restraint is used carefully, appropriately, and with respect.

## Appendix 1 Interview guide

**A short introductory text that is read to the respondents:** We know that restraint is used in neurosurgical care. No study has been conducted that has described nurses' experience of using restraint in neurosurgical care and we would therefore, like to study that. The questions I will ask you today are about your experience and your thoughts on restraint in your work as a nurse at neurosurgical department.

Background data

Sex

Age

Respondents’ workplaces

Experience as a nurse (years)

Experience as a nurse at a neurosurgical department (years)

1. Can you describe if there are any particular situations where restraint often occur?

Probing questions:

 a. What are the patients most often cared for and what are the circumstances?

 b. How do you feel about using restraint?

 c. Can you please describe some different types of restraint measures and describe how you feel about these?

d. Can you please describe the decision-making process?

e. Can you please describe reasons for using restraint?

2. Can you please describe a situation when you have used restraint during a shift at the neurosurgical department you work at?

Probing questions:

a. What was the patient cared for, and what were the circumstances surrounding the patient?

b. What were your thoughts during the incident?

c. Can you please describe the decision-making process?

d. When you think back on the situation, how do you think about the situation today?

3. Can you please describe another a situation when you have used restraint during a shift at the neurosurgical department you work at?

Probing questions:

a. What was the patient cared for, and what were the circumstances surrounding the patient?

b. What were your thoughts during the incident?

c. Can you please describe the decision-making process?

d. When you think back on the situation, how do you think about the situation today?

Ending:

I have now asked all the questions I intended to ask during the interview, is there anything you would like to comment on or add about what we have talked about?
